# Gastrointestinal bleeding in critically ill immunocompromised patients

**DOI:** 10.1186/s13613-021-00913-6

**Published:** 2021-08-21

**Authors:** Jennifer Catano, Sophie Caroline Sacleux, Jean-Marc Gornet, Marine Camus, Naike Bigé, Faouzi Saliba, Elie Azoulay, Guillaume Dumas, Lara Zafrani

**Affiliations:** 1 Intensive Care Unit, Saint-Louis Hospital, Assistance Publique Des Hôpitaux de Paris, Paris, France; 2 Intensive Care Unit, Paul Brousse Hospital, INSERM N°1193, Assistance Publique Des Hôpitaux de Paris, Université Paris-Saclay, Villejuif, France; 3 Gastroenterology Department, Saint-Louis Hospital, Assistance Publique Des Hôpitaux de Paris, Paris, France; 4 Department of Digestive Endoscopy, Saint-Antoine Hospital, Sorbonne Université, INSERM, Centre de Recherche Saint-Antoine and Assistance Publique-Hôpitaux de Paris, Paris, France; 5 Intensive Care Unit, Saint-Antoine Hospital, Assistance Publique Des Hôpitaux de Paris, Paris, France; 6 INSERM UMR976, Paris University, Paris, France

**Keywords:** Gastrointestinal bleeding, Intensive care unit, Immunocompromised host, Lymphoma

## Abstract

**Background:**

Acute gastrointestinal bleeding (GIB) may be a severe condition in immunocompromised patients and may require intensive care unit (ICU) admission. We aimed to describe the clinical spectrum of critically ill immunocompromised patients with GIB and identify risk factors associated with mortality and severe GIB defined by hemorrhagic shock, hyperlactatemia and/or the transfusion of more than 5 red blood cells units. Finally, we compared this cohort with a control population of non-immunocompromised admitted in ICU for GIB.

**Results:**

Retrospective study in 3 centers including immunocompromised patients with GIB admitted in ICU from January, 1st 2010 to December, 31rd 2019. Risk factors for mortality and severe GIB were assessed by logistic regression. Immunocompromised patients were matched with a control group of patients admitted in ICU with GIB. A total of 292 patients were analyzed in the study, including 141 immunocompromised patients (compared to a control group of 151 patients). Among immunocompromised patients, upper GIB was more frequent (73%) than lower GIB (27%). By multivariate analysis, severe GIB was associated with male gender (OR 4.48, CI95% 1.75–11.42, *p* = 0.00), upper GIB (OR 2.88, CI95% 1.11–7.46, *p* = 0.03) and digestive malignant infiltration (OR 5.85, CI95% 1.45–23.56, *p* = 0.01). Conversely, proton pump inhibitor treatment before hospitalization was significantly associated with decreased risk of severe GIB (OR 0.25, IC95% 0.10–0.65, *p* < 0.01). Fifty-four patients (38%) died within 90 days. By multivariate analysis, mortality was associated with hemorrhagic shock (OR 2.91, IC95% 1.33–6.38, *p* = 0 .01), upper GIB (OR 4.33, CI95% 1.50–12.47, *p* = 0.01), and long-term corticosteroid therapy before admission (OR 2.98, CI95% 1.32–6.71, *p* = 0.01). Albuminemia (per 5 g/l increase) was associated with lower mortality (OR 0.54, IC95% 0.35–0.84, *p* = 0.01). After matching with a control group of non-immunocompromised patients, severity of bleeding was increased in immunocompromised patients, but mortality was not different between the 2 groups.

**Conclusion:**

Mortality is high in immunocompromised patients with GIB in ICU, especially in patients receiving long term corticosteroids. Mortality of GIB is not different from mortality of non-immunocompromised patients in ICU. The prophylactic administration of proton pump inhibitors should be considered in this population.

**Supplementary Information:**

The online version contains supplementary material available at 10.1186/s13613-021-00913-6.

## Background

Acute gastrointestinal bleeding (GIB) may be a severe condition in immunocompromised patients and may require intensive care unit (ICU) admission. Data are scarce on GIB in immunocompromised patients and it relies mainly on case reports. Immunocompromised patients are at high risk of GIB for several reasons: first, coagulopathy is frequent in onco-hematological patients. Second, the GI tract is one of the most common primary sites of extra-nodal lymphomas and may occur in up to 20% of patients with non-Hodgkin’s lymphomas [1, 2]. Third, immunocompromised patients are at high risk of infections that may sometimes involve the GI tract and sometimes lead to GIB, such as cytomegalovirus (CMV) infection [3], herpes simplex virus (HSV) infection [4], aspergillosis [5], mucormycosis [6], mycobacteria [7] or clostridium difficile colitis [8].

Osman et al. have published recommendations in 2012 on the management by the intensivists of acute GIB in ICU [9]. However, recommendations rely mainly on studies published in unselected patients, whether they are immunocompromised or not. Studies focusing on immunocompromised patients are lacking.

In the present study, we aimed to describe the clinical spectrum of critically ill immunocompromised patients with GIB, ICU admission conditions, biological characteristics, endoscopic findings and outcomes. We aimed to identify risk factors associated with mortality and severe hemorrhage in this population. Finally, we compared this cohort with a control population of patients admitted in ICU for GIB.

## Methods

This study was approved by an Institutional Review Board (Comité d’Éthique de la Société de Réanimation de Langue Française #CE-SRLF 19-55), according to the French regulation on non-interventional studies, which waived the need for signed informed consent for patients included in this database. No data allowing identification of the patients included in the study were recorded. The study was conducted in accordance with the Declaration of Helsinki principles.

### Design and setting

**Fig. 1:**
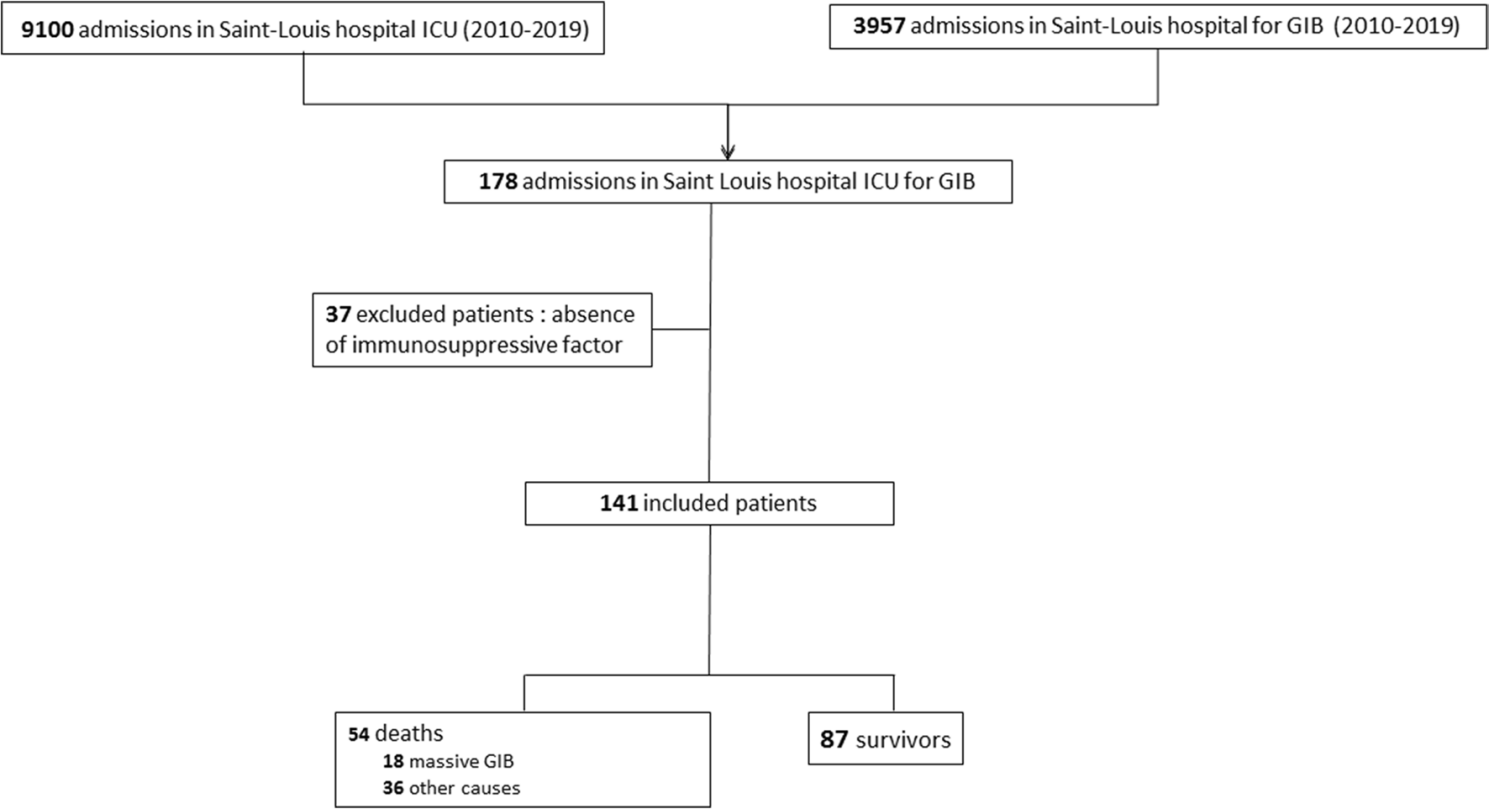
Flow chart

We included consecutive immunocompromised adults admitted to our ICU in the Saint-Louis University Hospital, Paris, France, between January, 1st 2010 and December, 31^rd^ 2019, with GIB. During this period, 3957 patients who were hospitalized experienced GIB. Among them, 178 patients (4.5%) required ICU admission (Fig. 1). Immunosuppression was defined as use of long-term (> 3 months) or high-dose (> 0.5 mg/kg/day) steroids, use of other immunosuppressant drugs, solid organ transplantation, solid tumor requiring chemotherapy in the last 5 years, hematological malignancy regardless of time since diagnosis and received treatments, or primary immune deficiency**.** The Saint-Louis University Hospital is a 700-bed public hospital with 330 beds for patients with hematological malignancies and solid cancers. The ICU is a 16-bed medical unit that admits 1000 patients per year, of whom about three quarters of them are immunocompromised. Information on the organization of our ICU and criteria for ICU admission has been published elsewhere [10]. For the control group (151 patients before matching), non-immunocompromised patients with GIB hospitalized in Paul Brousse University hospital and Saint-Antoine University hospital between August, 1st 2017 and April, 31rd 2020 were included. Among the 151 control patients, 65 patients had cirrhosis and 86 patients did not have cirrhosis.

#### Clinical, laboratory and endoscopic assessment

ICU medical records of selected patients were then reviewed for age, sex, underlying diseases, clinical and biological presentation at ICU admission, the need for organ support, endoscopic findings, transfusions, and outcome. For each patient, data were recorded at the time of initial evaluation of GIB at the time of ICU admission, and during ICU stay. Etiological diagnoses were reviewed. Specific malignant etiologies were confirmed by histological findings, infectious etiologies were confirmed by specific germs identification. Transfusion data were retrieved from the French Blood Establishment database. Transfusion policy followed the guidelines in non-hematological patients using fresh frozen plasma (FFP), platelet concentrates (PC) and red blood cells (RBC) units with a 1:1:2 target ratio for a hemoglobin target of 7 to 9 g/dl [11]. Endoscopic parameters included the bleeding location and interventions (hemostatic management or biopsy). Other hemostatic gestures were recorded, such as arterio-embolization or need for hemostatic surgery.

### Definitions

The Sequential Organ Failure Assessment (SOFA) score was also recorded, as previously defined [11]. Septic shock was defined according to the Sepsis-3 consensus definition [12]. Hemorrhagic shock was defined by excessive bleeding associated with arterial hypotension resulting in the use of vasopressors and/or excessive bleeding associated with lactatemia > 2 mmol/l. Severe GIB in our ICU cohort was defined as hematemesis, hematochezia, or melena coupled with hemorrhagic shock and/or lactatemia > 2 mmol/l and /or GI bleeding that require the transfusion of more than 5 units of RBC (5 units corresponds to the median of RBCs that has been transfused per patient in our cohort).

The AIMS65 score assigns 1 point for each of the following: albumin level < 30 g/L, international normalized ratio > 1.5, altered mental status, systolic blood pressure < 90 mm Hg, and age older than 65 years. Severe GIB according to the AIMS65 score was defined as an AIMS65 score ≥ 2 [13].

GIB is classified as upper GIB if the site of hemorrhage is proximal to the ligament of Treitz or as lower GIB if the site of hemorrhage is distal to the ligament of Treitz.

### Statistical analysis

Continuous variables are described as median and interquartile range (IQR) and compared using Wilcoxon’s rank sum test; categorical variables are summarized by counts (percents) and compared using exact Fisher test. Day-90 mortality and severe GIB (as defined above) were analysed as a binary variable.

First, factors associated with outcomes were assessed using multivariate analysis by logistic regression. Variables achieving *p* < 0.20 in univariate analyses were entered into the multivariate logistic regression model. A multiple backward-stepwise selection procedure eliminated those variables with an exit threshold set at *p* = 0.05, after testing for collinearity between variables and checking the assumption of log-linearity. Goodness of fit was evaluated using Le Cessie–van Houwelingen’s method and discrimination with C-statistic.

Thereafter, to assess the prognostic effect of an immunocompromised status in the context of an observational cohort, a matched comparison with control patients was performed. Immunocompromised patients were individually matched in a 1:1 ratio to a control group of non-immunocompromised patients. The 5 matching criteria were: age (exact match), hemoglobin (exact match), bleeding location (classified as upper or lower bleeding; exact match), SOFA score (exact match) and proton pump inhibitor preventive treatment (exact match). We used logistic regression models with a random effect on matching clusters to understand association between immunocompromised status, patient’s characteristics and outcome.

The measures of associations are presented with odds ratios and confidence intervals at 95%. All tests were two-sided and *p* values lower than 5% were considered to indicate significant associations. Analyses were performed using R statistical platform, version 3.0.2 (https://cran.r-project.org/).

## Results

### Characteristics of immunocompromised patients with GIB

One hundred and forty-one immunocompromised patients were included in the study (Fig. 1). Median age was 60 years (IQR 48–69), and 89 patients (63%) were men. Patients’ characteristics are shown in Table 1 and Additional file 1: Table S1. One hundred and six patients (75%) were already hospitalized before ICU admission, with a median length of stay of 12 [4–25] days. Hematological malignancy was the main cause of immunosuppression, diagnosed in 93 (66%) patients including 41 lymphomas (29%), 17 acute leukemias (12%), 16 myelomas (11%), 5 chronic leukemias (4%) and 6 myelodysplasias (4%). Sixty-one patients (43%) had received chemotherapy in the last 6 months for solid tumor or hematological malignancy.

**Table 1:** Immunocompromised patients’ characteristics

Characteristics	Patients *N* = 141
Generalities	
Age, median [IQR]	60 [48–69]
Male gender, *n* (%)	89 (63)
BMI, median [IQR]	24 [21–29]
Immunosuppression factor	
Hematological malignancy, *n* (%)	93 (66)
Auto-immune disease, *n* (%)	17 (12)
Solid organ transplant, *n* (%)	17 (12)
HIV, *n* (%)	24 (17)
Corticosteroids, *n* (%)	72 (51)
Chemotherapy, *n* (%)	61 (43)
Other immunosuppressive drugs, *n* (%)	37 (26)
Allograft, *n* (%)	9 (6)
Treatments at admission	
Anticoagulants, *n* (%)	36 (26)
Antiplatelet agent, *n* (%)	21 (15)
PPI before admission, *n* (%)	69 (49)
GIB characteristics	
Upper GIB, *n* (%)	103 (73)
Delay ICU—GIB, median [IQR], days	0 [− 2.1 to 1.25]
Melena, *n* (%)	90 (64)
Hematochezia, *n* (%)	51 (36)
Hematemesis, *n* (%)	42 (30)
Biology during GIB	
Thrombopenia < 50 G/L, *n* (%)	56 (40)
Hemoglobin (nadir) (g/dl), median [IQR]	6,6 [2, 5–7]
PT (%), median [IQR]	69 [57–77.5]
ACT, median [IQR]	1.15 [1–1.5]
Lactatemia (mmol/l), median [IQR]	2.1 [1.2–3.9]
Fibrinogen (g/l), median[IQR]	3.095 [2.062–4.825]
Organ failure	
Shock, *n* (%)	64 (45)
Vasopressive drugs, *n* (%)	55 (39)
Renal replacement therapy, *n* (%)	44 (31)
Mechanical ventilation, *n* (%)	110 (79)
SOFA score during GIB, median [IQR]	6 [3–12]
Transfusion	
RBC units, median [IQR]	5 [3–10]
Platelets units, median [IQR]	6 [0–19]
FFP units, median [IQR]	0 [0–2]
Fibrinogen concentrates, *n* (%)	7 (5)
Tranexamic acid, *n* (%)	5 (4)
Etiologies	
Ulcers, any cause, *n* (%)	38 (27)
Malignant lesion, *n* (%)	36 (26)
Variceal bleeding, *n* (%)	15 (11)
Infectious cause, *n* (%)	13 (9)
Angiodysplasia, *n* (%)	8 (6)
Noninfectious colitis, *n* (%)	8 (6)
Diverticular hemorrhage, *n* (%)	4 (3)
Graft versus host disease, *n* (%)	2 (1)
Other, *n* (%)	17 (12)
Interventional care	
EGD, hemostatic procedure, *n* (%)	59 (42)
Colonoscopy, *n* (%)	42 (30)
CT angiography, *n* (%)	34 (24)
Arterio-embolization, *n* (%)	10 (7)
Hemostatic surgery, *n* (%)	10 (7)
AIM65 score ≥ 2, *n* (%)	105 (74)
ICU stay, median [IQR], days	5 [2–11]
Relapses, *n* (%)	38 (27)
ICU mortality, *n* (%)	30 (21)
Mortality at day 30, *n* (%)	39 (28)
Mortality at day 90, *n* (%)	54 (38)
Mortality due to GIB, *n* (%)	18 (13)

*BMI* body mass index, *HIV* human immunodeficiency virus, *PPI* proton pump inhibitor, *ICU* intensive care unit, *GIB* gastrointestinal bleeding, *PT* prothrombin time, *ACT* activated coagulation time, *SOFA* sequential organ failure assessment, *RBC* red blood cell, *FFP* fresh frozen plasma, *GVH* graft versus host, *EGD* esophagogastroduodenoscopy, *CT* computed tomography

Before ICU stay, a proton pump inhibitor (PPI) preventive treatment had been prescribed in almost half of the patients (49%, *n* = 69), 26% of the patients (*n* = 36) were treated with anticoagulant therapy and 21 patients (15%) with antiplatelet therapy. Biological results are listed in Table 1. The median albumin level during GIB was 26 g/l [22–30].

Median ICU stay was 5 days (IQR 2–11). Median SOFA score during GIB was 6 [3–12]. Sixty-four patients (45%) presented with hemorrhagic shock, 51 patients (36%) required mechanical ventilation because of respiratory failure and 44 patients (31%) required renal replacement therapy during ICU stay.

Red blood cell (RBC) transfusion was required in 130 patients (92%) with a median number of RBC units of 5 per patient [3–10]. Platelet transfusion was required in 84 patients (60%) with a median number of 6 [0–19] units per patient. Finally, FFP was transfused in 51 patients (36%).

### Characteristics of GIB in immunocompromised patients

At GIB presentation, 90 patients (64%) presented with melena, 51 patients (36%) with hematochezia and 42 patients (30%) with hematemesis before ICU admission or during ICU stay. Upper GIB was more frequent (*n* = 103, 73%) than lower GIB (*n* = 38, 27%). Most patients were treated with continuous intravenous PPIs (72%, *n* = 97). Esophagogastroduodenoscopy (EGD) was performed for 123 patients (87%) with 59 endoscopic hemostatic procedures (25 clips, 41 epinephrine injections, 11 sclerotherapies, 19 electrocoagulations, 9 hemostatic spray). Fifteen patients needed at least 3 EGD to control hemorrhage. Colonoscopy was performed in 42 patients (30%) with 8 hemostatic procedures. Finally, 19 patients (13%) presented with refractory GIB with the need for arterio-embolization in 9 patients, surgical hemostasis in 9 patients. One patient required both techniques.

Etiologies of GIB are listed in Table 1. The most frequent cause of GIB was gastroduodenal ulcer diagnosed in 38 patients (27%). Malignant lesions (specific digestive infiltration of the underlying malignancy) were diagnosed in 36 patients (26%). Twenty-seven of them were lymphoma specific lesions, 4 solid tumors, one amyloidosis and one Kaposi sarcoma. Among infectious etiologies (*n* = 13, 9%), 5 patients had gastrointestinal CMV, 4 had Clostridium difficile colitis, 2 had Candida esophagitis, 1 had invasive intestinal aspergillosis and 1 patient had invasive adenovirus infection. Noninfectious colitis included neutropenic enterocolitis, ischemic colitis or toxic colitis. Portal hypertension in patients with variceal bleeding was secondary to primary sclerosing cholangitis in 5 cases, neoplastic portal vein obstruction in 8 cases (including 6 veno-occlusive diseases) and Budd Chiari syndrome in 2 cases.

### Outcomes of immunocompromised patients with GIB

**Fig. 2:**
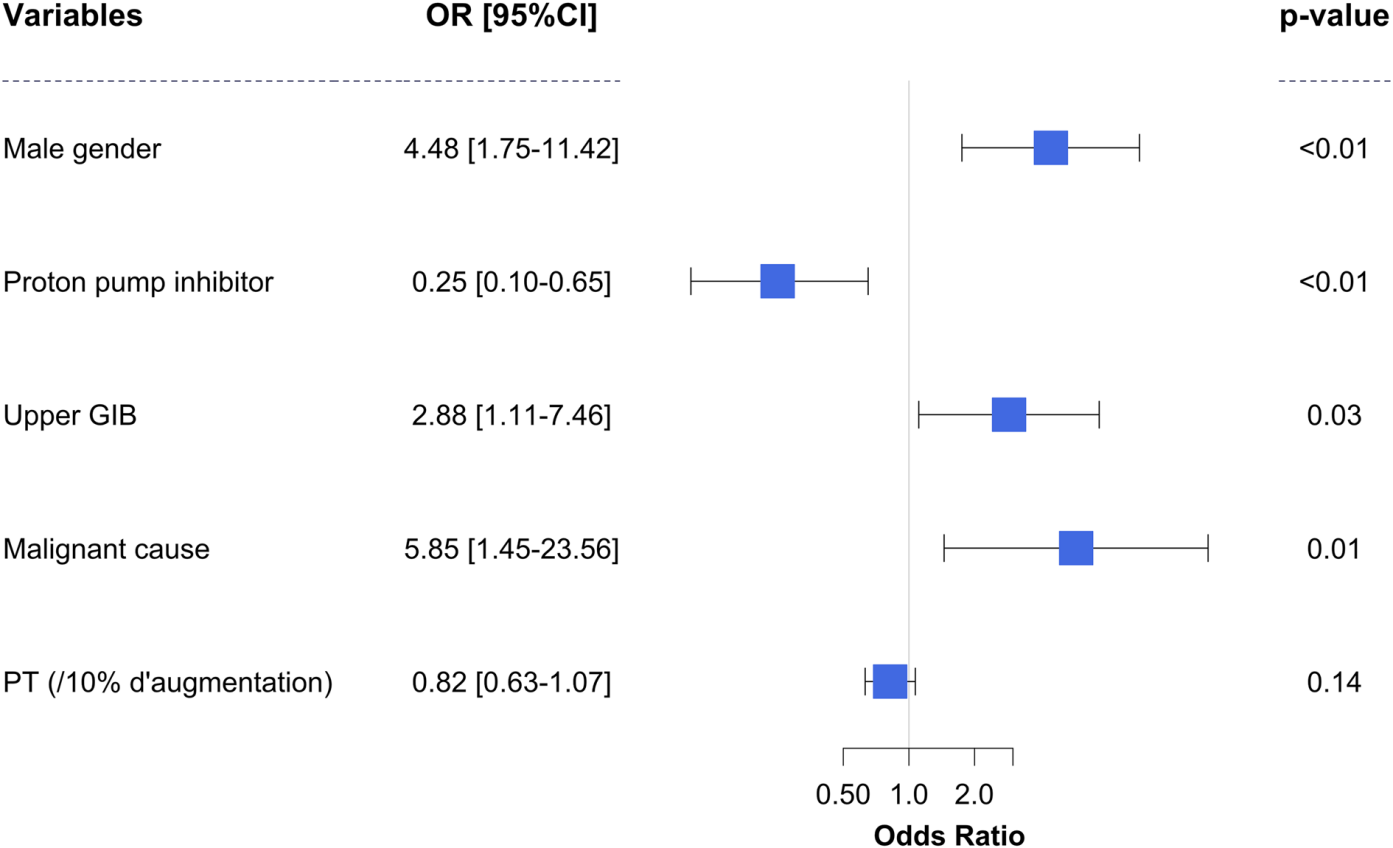
Factors associated with severe gastrointestinal bleeding by multivariate analysis. *GIB* gastrointestinal bleeding, *PT* prothrombin time

One hundred and four patients (74%) presented with severe GIB. Univariate analysis of risk factors associated with severe GIB is shown in Additional file 1: Table S2. By multivariate analysis, severe GIB was associated with male gender (OR 4.48, CI95% 1.75–11.42, *p* 0.00), upper GIB (OR 2.88, CI95% 1.11–7.46, *p* = 0.03) and digestive malignant infiltration (OR 5.85, CI95% 1 0.45–23.56, *p* = 0.01) (Fig. 2). Conversely, PPI treatment before hospitalization was significantly associated with decreased risk of severe GIB (OR 0.25, CI95% 0.10–0.65, *p* < 0.01). Interestingly, the protective effect of PPI treatment remained significant when GIB related to ulcers were removed (OR 0.22 CI95% 0.06–0.70, *p* = 0.02) and if lower GIB were excluded from this analysis (OR 0.13, CI95% 0.01–0.67, *p* = 0.13).

**Fig. 3:**
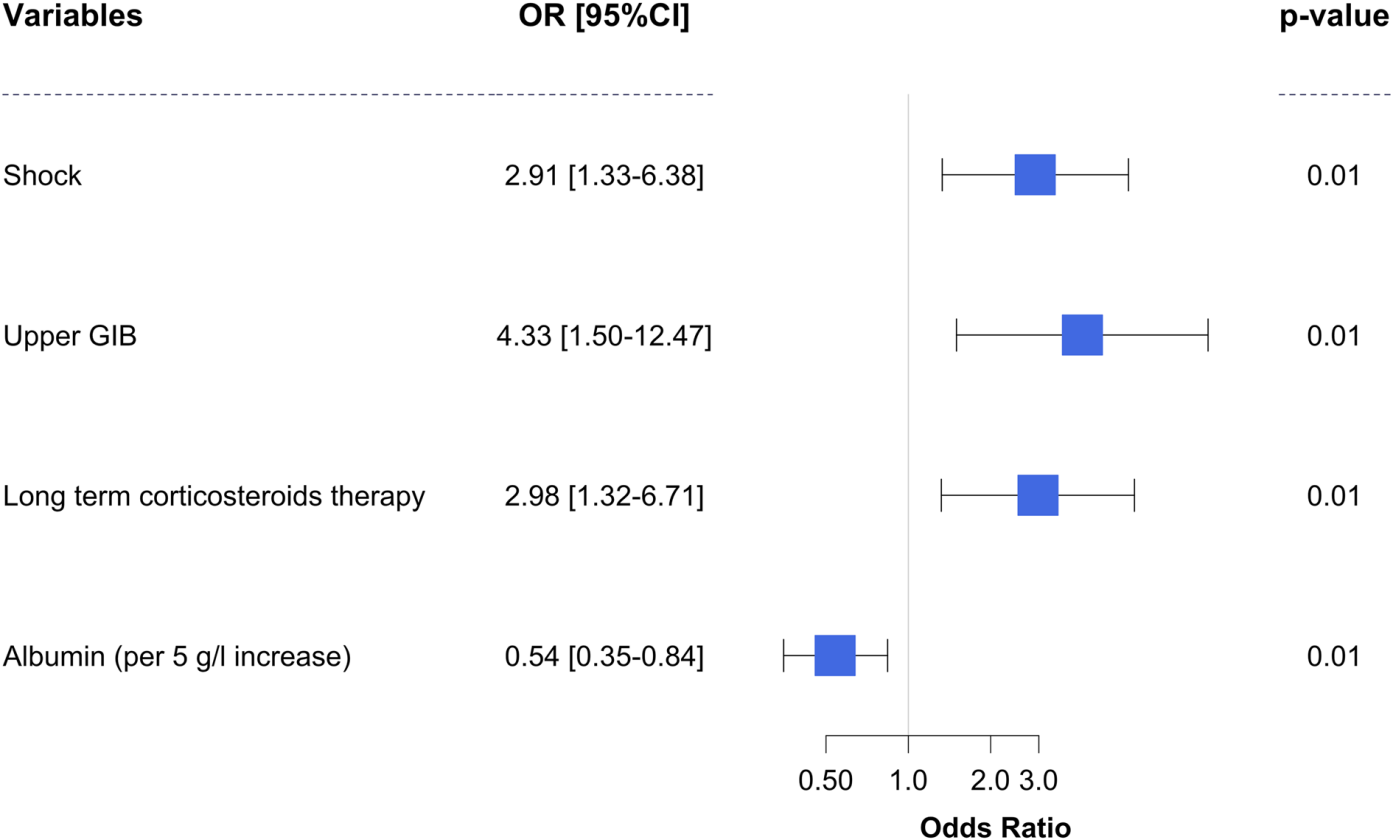
Factors associated with mortality by multivariate analysis. *GIB* gastrointestinal bleeding

Fifty-four patients (38%) died within 90 days. Univariate analysis of risk factors associated with mortality is shown in Additional file 1: Table S3. By multivariate analysis, mortality was associated with hemorrhagic shock (OR 2.91, CI95% 1.33–6.38, *p* = 0 0.01), upper GIB (OR 4.33, CI95% 1.50–12.47, *p* = 0.01), and long-term corticosteroid therapy before admission (OR 2.98, CI95% 1.32–6.71, *p* = 0.01) (Fig. 3). Albuminemia (per 5 g/l increase) was associated with lower mortality (OR 0.54, CI95% 0.35–0.84, *p* = 0.01). AIM65 score was not associated with mortality (OR 2.26, CI95% 0.83–6.18, *p* 0.11, Additional file 1: Table S4).

### Comparison of outcomes of immunocompromised patients and non-immunocompromised patients with GIB

One hundred and fifty one non-immunocompromised critically ill patients were included in the control group before matching. Among them, there were 65 (43%) cirrhotic patients with a median Child score at admission at 12 [11–13] including 56 patients (37%) with an acute-on-chronic liver failure (ACLF). After statistical matching by age, hemoglobin levels, location of GIB, SOFA score and proton pump inhibitor preventive treatment, 112 patients in each group were compared (Additional file 1: Figure S1). Characteristics of the 2 groups are shown in Table 2.

**Table 2:** Case/control comparison after matching

Characteristics	Case group		Control group		*p* value
	*n* = 112	% or med [IQR]	*n* = 112	% or med [IQR]	
Generalities					
Age	62	[48–70]	60	[50–69]	0.92
Male gender	85	76%	70	62%	0.042
BMI	26	[24–29]	24	[21–28]	0.11
Treatments at admission					
Anticoagulants	15	13%		21%	0.88
Antiplatelet agent	14	12%	16	14%	0.26
PPI before admission	44	39%	47	42%	0.79
GIB characteristics					
Upper GIB	93	83%	89	79%	0.61
Melena	66	59%	72	64%	0.49
Hematochezia	27	24%	37	33%	0.18
Hematemesis	50	45%	38	34%	0.13
Biology during GIB					
Thrombopenia < 50 G/L	13	19%	41	37%	0.0.18
Hemoglobin (nadir) (g/dl)	6.4	[5.8–7.2]	6.7	[5.9–7.4]	0.47
PT (%)	48	[33–75]	70	[57–78.5]	< 0.0001
ACT	1.65	[1.34–2.4]	1.14	[1–1.5]	< 0.0001
Lactatemia (mmol/l)	1.7	[1–3.3]	2.1	[1.2–3.9]	0.22
Fibrinogen < 1 g/l	15	15%	4	4%	0.008
Organ failure					
Shock	47	42%	48	43%	1.00
Vasopressive drugs	47	42%	40	36%	0.41
Renal replacement therapy	27	24%	32	29%	0.54
Mechanical ventilation	96	86%	86	77%	0.12
SOFA score during GIB	6	[4–11]	6	[3–12]	0.37
AIM 65	2	[1–3]	2	[1–3]	0.24
AIM65 score ≥ 2, *n* (%)	83	74%	74	66%	0.24
Transfusion					
RBC units	4	[2–7]	5	[2–9]	0.11
Platelets units	0	[0–0]	5.5	[0–18]	< 0.0001
FFP units	0	[0–2]	0	[0–2]	0.76
Interventional care					
EGD, hemostatic procedure	75	68%	52	46%	0.002
Colonoscopy	22	20%	31	28%	0.21
CT angiography	13	12%	20	18%	0.26
Arterio-embolization	7	6%	7	6%	1.00
Hemostatic surgery	3	3%	6	5%	0.50
Length of ICU stay*, days*	4	[2–15]	5	[2–10]	0.085
Length of hospital stay, *days*	32	[12–58]	29	[13–47]	0.72

*BMI*: body mass index, *ACLF* acute-on-chronic liver failure, *PPI* proton pump inhibitor, *ICU* intensive care unit, *GIB* gastrointestinal bleeding, *PT* prothrombin time, *ACT* activated coagulation time, *SOFA* sequential organ failure assessment, *RBC* red blood cell, *FFP* fresh frozen plasma, *GVH* graft versus host, *EGD* esophagogastroduodenoscopy, *CT* computed tomography, *TIPS* transjugular intrahepatic portosystemic shunts

After matching, there was no difference in terms of ICU mortality, mortality at day 30 or at day 90 between immunocompromised and non-immunocompromised patients. However, GIB was associated with increased severity of bleeding in immunocompromised patients when compared to controls (Tables 3, 4).

**Table 3:** Number of events case–control after matching

	ICU mortality	Mortality at day 30	Mortality at day 90	Severe GIB
Immunosuppressed group (case) (*n*, %)	26 (23)	33 (29)	41 (36)	86 (76)
Control group (*n*, %)	28 (25)	28 (25)	37 (33)	68 (60)

**Table 4:** Comparison case–control after matching

	OR [CI 95%]	*p* value
ICU mortality	0.90 [0.49–1.68]	0.75
Mortality at day 30	1.25 [0.69–2.25]	0.46
Mortality at day 90	1.17 [0.67–2.05]	0.57
Severe GIB	2.13 [1.17–3.85]	0.01

## Discussion

This study is the largest cohort published to date focusing on GIB in immunocompromised patients in ICU. We found that the severity of GIB was associated with upper location of hemorrhage and a specific digestive infiltration of the underlying malignancy. The administration of PPIs before GIB was associated with reduced risk of severe GIB. Upper location of hemorrhage, shock, hypoalbuminemia and corticosteroids were associated with mortality. Finally, although the severity of GIB in immunocompromised patients is higher than non-immunocompromised patients, mortality is not different between the 2 groups.

Data comparing mortality in patient with upper location of GIB or lower location of GIB are conflicting [14]. In accordance with our results, Lanas et al*.* found that the number of transfused RBC units was higher in upper GIB than lower GIB [15]. The cause of upper GIB is crucial in the interpretation of this result. In patients with endoscopically confirmed tumor bleeding, endoscopic hemostasis may be difficult and re-bleeding is frequent. Indeed, in a cohort of 71 patients with tumor bleeding, Schatz et al*.* showed that re-bleeding was common and mortality was high [16]. To date, there are no specific recommendations for endoscopic therapies in patients with tumor bleeding. Data generally have failed to show superiority of one method over another, and each may be useful depending on location of the bleeding source and patient characteristics. In case of tumor hemorrhage, powder spray such as Hemospray (Cook Medical, Winston-Salem, North Carolina, USA), are increasingly used allowing access to lesions in difficult locations and treatment of larger surface areas than others devices [20]. In the case of failure of endoscopic hemostasis, transcatheter angiographic embolization should be considered.

Another important finding in our study is the association between hypoalbuminemia and mortality. This finding is in accordance with previous studies that have demonstrated the role of hypoalbuminemia in predicting mortality in upper GIB, including in critically ill patients [17]. Albuminemia has been included in the AIMS65 score that was developed by Saltzmann et al. who showed that AIMS65 score was an accurate risk score to predict in-hospital mortality [13]. If hypoalbuminemia has been related to cirrhosis in previous studies, we show here that hypoalbuminemia is also an independent factor of mortality in immunocompromised patients without cirrhosis. However, we did not found any correlation between AIMS65 score and mortality in our cohort. Indeed, the AIMS65 score is a risk stratification score validated to predict in-hospital mortality in patients presenting with GIB. This score is useful in the emergency department to predict patients who will require ICU [13]. As we focused our study on patients who were already hospitalized in ICU, most of them (74%) had an elevated AIMS65 score at admission. This score may then be not appropriate to define severe GIB in ICU.

The association between corticosteroids and mortality in our study raises some questions. In a previous systematic review with meta-analysis of trials including both ambulatory and hospitalized patients, an increased incidence of GIB of any severity was observed with corticosteroids [18]. Recently, Butler et al*.* performed a large systematic review with meta-analysis of randomized control studies involving adult critically ill patients and found that corticosteroids versus placebo or no treatment may have increased the risk of clinically important GIB, but not GIB of any severity [19]. Unfortunately, although our patients received high doses of corticosteroids because of their underlying pathology, we were not able to compare the doses and duration of administration of corticosteroids to predict the risk of GIB according to a corticosteroids dose threshold. Whether corticosteroids are a marker of severity of our patients, or a factor directly contributing to severe GIB is still unknown.

The role of PPIs to prevent ulcer in the ICU is still an ongoing debate. Krag et al. in a multicenter European trial in ICU patients who were at risk for GIB did not found any difference in terms of mortality and number of clinically important events in patients who were assigned to pantoprazole and those assigned to placebo. However, the incidence of GIB in this trial is very low: clinically important GIB occurred in 2.5% of the patients in the pantoprazole group and in 4.2% of the patients in the placebo group [20]. In a recent meta-analysis, Wang et al. found that PPIs have no impact on mortality but probably reduce clinically important bleeding [21]. Similarly, we found that PPI administration before GIB was associated with less severe hemorrhage. Osman et al. recommend stress ulcer prophylaxis in patients with a history of peptic ulcer, and/or in patients receiving antiplatelet therapy, and/or in ventilated patients in the absence of enteral feeding and/or in patients with coagulopathy [9]. In a recent meta-analysis, Granholm et al. identified predictors of GIB in ICU patients and found that coagulopathy, acute kidney injury, shock and chronic liver disease were associated with important GIB [22]. Most of our patients were at high risk of GIB before ICU admission because of coagulopathies due to hematological malignancies or chemotherapies. However, only 50% of them had received PPIs prior to GIB.

Finally, the comparison between our cohort and a control cohort did not show any difference in terms of mortality between immunocompromised patients with GIB and non-immunocompromised patients, after matching for severity (SOFA score). We found a mortality of 38% at 90 days in our cohort. Of note, in the control group, after matching, 49 (44%) patients had cirrhosis. Mortality is known to be high in cirrhotic patients admitted in ICU and associated with organ failures [23], up to 42% in cirrhotic patients admitted with GIB and ACLF in recent series [24]. It is also important to notice that only 18 (13%) of immunocompromised patients died because of GIB in our cohort and 30 patients died in ICU (21%). The high mortality of immunocompromised patients is then also related to the severity of their underlying diseases.

This study has several limitations. First, because of the retrospective design of the study, unidentified confounding factors may have been overlooked in the multivariable analysis. Second, many patients did not undergo endoscopic biopsies allowing a definite pathological diagnosis. Third, as heart rates were not available, we may have missed some patients with hemorrhagic shock according to literature standards [11]. Moreover, our study did not have the power to compare the efficiency of the different hemostatic strategies. Finally, outcomes of patients with lower or upper GIB who were hospitalized but did not require ICU admission were not available and would have provided valuable information.

## Conclusion

Mortality is high in immunocompromised patients with GIB in ICU and not different from mortality of non-immunocompromised patients with GIB in ICU. Severity is associated with upper location of GIB and the presence of specific digestive tumor infiltration. Upper location of hemorrhage, shock, hypoalbuminemia and corticosteroids are associated with mortality. The prophylactic administration of PPIs should be considered in this population.

## Supplementary Information

**Additional file 1: Table S1.** Immunocompromised patients’ characteristics according to GIB localization. **Table S2.** Univariate analysis: factors associated with severe gastrointestinal bleeding. **Table S3.** Univariate analysis: risk factors associated with mortality. **Table S4.** Factors associated with mortality by multivariate analysis (including the AIM65 score). **Table S5.** Factors associated with mortality by multivariate analysis (including the FFP/RBC ratio). **Figure S1.** Case–control analysis: Love plot comparing SMD (standardized Mean Differences) before and after matching.

## Data Availability

The datasets used and/or analyzed during the current study are available from the corresponding author on reasonable request.

## Abbreviations

ACLF: Acute-on-chronic liver failure
AIMS65 score: Albumine, INR, mental status
CMV: Cytomegalovirus
CE-SRLF: Comité d’Éthique de la Société de Réanimation de Langue Française
EGD: Esophagogastroduodenoscopy
FFP: Fresh frozen plasma
GIB: Gastrointestinal bleeding
GVH: Graft versus host disease
HIV: Human immunodeficiency virus
HSV: Herpes simplex virus
ICU: Intensive care unit
IQR: Interquartile range
OR: Odds ratio
PPI: Proton pump inhibitor
RBC: Red blood cells
SOFA: Sequential Organ Failure Assessment
