# Supplementary material for: Gastrointestinal bleeding in critically ill immunocompromised patients
Source: Ann Intensive Care. 2021 Aug 21;11:130. doi: 10.1186/s13613-021-00913-6 (PMC8380218; doi:10.1186/s13613-021-00913-6)
Supplement: Supplementary file 1 — Additional file 1: Table S1. Immunocompromised patients’ characteristics according to GIB localization. Table S2. Univariate analysis: factors associated with severe gastrointestinal bleeding. Table S3. Univariate analysis: risk factors associated with mortality. Table S4. Factors associated with mortality by multivariate analysis (including the AIM65 score). Table S5. Factors associated with mortality by multivariate analysis (including the FFP/RBC ratio). Figure S1. Case–control analysis: Love plot comparing SMD (standardized Mean Differences) before and after matching. [file 13613_2021_913_MOESM1_ESM.docx]

**Table S1 Immunocompromised patients’ characteristics according to GIB localization**

| **Characteristics** | **Patients**  **n=141** | **Upper GIB**  **N = 103** | **Lower GIB**  **N=38** | **P value** |
| --- | --- | --- | --- | --- |
| **Generalities**  Age, *median [IQR]*  Male gender, *n (%)*  Weight, *median [IQR]*  BMI, *median [IQR]* | 60 [48 - 69]  89 (63)  70,5 [61,2 – 83]  24 [21 – 29] | 59 [49-69]  65 (63)  70 [61.5-83]  24 [21.75-29] | 61[38-66]  24 (63)  73 [63-83.5]  24 [20.75-29] | 0.33  1  0.93  0.59 |
| **Immunosuppression factor**  Hemopathy, *n (%)*  Auto-immune disease, *n (%)*  Solid organ transplant, *n (%)*  HIV, *n (%)*  Corticosteroids, *n (%)*  Chemotherapy, *n (%)*  Immunosuppressive drugs, *n (%)*  Allograft, *n (%)* | 93 (66)  17 (12)  17 (12)  24 (17)  72 (51)  61 (43)  37 (26)  9 (6) | 70 (68)  10 (10)  10 (10)  19 (18)  50 (49)  47 (46)  22 (21)  8 (8) | 23 (61)  7 (18)  7 (18)  5 (13)  22 (58)  14 (37)  15 (39)  1 (3) | 0.53  0.26  0.26  0.62  0.43  0.46  0.051  0.47 |
| **Treatments at admission**  Anticoagulants, *n (%)*  Antiplatelet agent, *n (%)*  PPI before admission, *n (%)*  **Treatments during ICU**  Enteral nutrition, *n (%)*  Intravenous nutrition, *n (%)* | 36 (26)  21 (15)  69 (49)  50 (36)  30 (23) | 25 (24)  16 (16)  50 (49)  38 (38)  24 (26) | 11 (29)  5 (13)  19 (50)  12 (32)  6 (18) | 0.78  0.9  1  0.72  0.49 |
| Delay ICU – GIB, *median [IQR], days* | 0 [-2.1 – 1.25] | 0 [-2-1.75] | 0 [-1-0.75] | 0.88 |
| **Biology during GIB**  Thrombopenia < 50 G/L, *n (%)*  Hemoglobin (nadir), *median [IQR]*  PT, *median [IQR]*  ACT, *median [IQR]*  Lactatemia, *median [IQR]*  Fibrinogen (g/l), *median[IQR]* | 56 (40)  6,6 [5,5– 7,2]  69 [57 – 77.5]  1.15 [1 – 1.5]  2.1 [1.2 – 3.9]  3.095 [2.062 – 4.825] | 43 (42)  6.45 [5.5-7.2]  68 [56-77]  1.18 [1-1.5]  2.4 [1.2-4.35]  3.1 [1.91-4.83] | 13 (34)  6.8 [6.1-7.5]  72 [58.75-79.75]  1.085 [1.03-1.48]  1.8 [1.25-2.4]  2.96 [2.32-4.21] | 0.51  0.18  0.27  0.65  0.098  0.57 |
| **Organ failure**  Shock, *n (%)*  Vasopressive drugs, *n (%)*  Renal Replacement Therapy, *n (%)*  Mechanical ventilation, *n (%)*  SOFA score, *median [IQR]* | 64 (45)  55 (39)  44 (31)  110 (79)  6 [3 – 12] | 52 (50)  47 (46)  34 (33)  85 (83)  6 [3-13.5] | 12 (32)  8 (21)  10 (26)  25 (66)  4 [2-7] | 0.07  0.012  0.58  0.044  0.058 |
| **Transfusion**  RBC units, *median [IQR]*  Platelets units, *median [IQR]*  FFP units, *median [IQR]*  Fibrinogen concentrates, *n (%)*  Tranexamic acid, *n (%)* | 5 [3 – 10]  6 [0 – 19]  0 [0 - 2]  7 (5)  5 (4) | 5 [3-10]  8 [0-20]  0 [0-3]  5 (5)  3 (3) | 5 [2.25-9.75]  2.5 [0-12.25]  0 [0-2]  2 (5)  2 (5) | 0.66  0.19  0.61  1  0.88 |
| **Etiologies**  Ulcers, any cause, *n (%)*  Malignant lesion, *n (%)*  Variceal bleeding, *n (%)*  Infectious cause, *n (%)*  Angiodysplasia, *n (%)*  Noninfectious colitis, *n (%)*  Diverticular hemorrhage, *n (%)*  Graft versus host disease, *n (%)*  Other, *n (%)* | 38 (27)  36 (26)  15 (11)  13 (9)  8 (6)  8 (6)  4 (3)  2 (1)  17 (12) | 38 (37)  22 (21)  15 (15)  7 (7)  6 (6)  0  0  0  15 (15) | 0  14 (37)  0  6 (16)  2 (5)  8 (6)  4 (3)  2 (1)  2 (5) | <0.0001 |
| **Interventional care**  EGD, hemostatic procedure, *n (%)*  Colonoscopy, *n (%)*  CT angiography, *n (%)*  Arterio-embolization, *n (%)*  Hemostatic surgery, *n (%)* | 59 (42)  42 (30)  34 (24)  10 (7)  10 (7) | 57 (55)  17 (17)  21 (20)  7 (7)  6 (6) | 2 (5)  25 (66)  13 (34)  3 (8)  4 (11) | <0.0001  <0.0001  0.14  1  0.55 |
| AIM65 score ≥ 2, n (%)  ICU stay, *median [IQR], days* | 105 (74)  5 [2 – 11] | 80 (78) | 25 (66) | 0.22 |
| Relapses, n (%)  ICU mortality, n(%)  Mortality at day 30, n(%) | 38 (27)  30 (21)  39 (28) | 26 (39)  27 (26)  35 (34) | 12 (43)  3 (8)  4 (11) | 0.89  0.033  0.011 |
| Mortality at day 90, n (%)  Mortality due to GIB, n (%) | 54 (38)  18 (13) | 48 (47)  14 (14) | 6 (16)  4 (11) | 0.002  0.11 |

*BMI: body mass index, HIV: human immunodeficiency virus, PPI: proton pump inhibitor, ICU: intensive care unit, GIB: gastrointestinal bleeding, PT: prothrombin time, ACT: activated coagulation time, SOFA: sequential organ failure assessment, RBC: red blood cell, FFP: fresh frozen plasma, GVH: graft versus host, EGD: esophagogastroduodenoscopy, CT: computed tomography.*

**Table S2: Univariate analysis: factors associated with severe gastrointestinal bleeding**

| **Characteristics** | No severe GIB | | Severe GIB | | | p value |
| --- | --- | --- | --- | --- | --- | --- |
|  | n=33 | % or med [IQR] | n=108 | % or med [IQR] | |  |
| **Generalities**  Age  Male gender  Weight  BMI | 61  14  65  23 | [54 – 72]  42 %  [55 – 70]  [19 – 25] | 57.5  75  73  25 | | [46.5 – 68]  69 %  [65.5 – 86]  [22 – 30] | 0.25  0.007  0.002  0.004 |
| **Immunosuppression factor**  Hemopathy  Auto-immune disease  Solid organ transplant  HIV  Corticosteroids  Chemotherapy  Immunosuppressive drugs  Allograft | 21  8  5  2  17  13  10  1 | 64 %  24 %  15 %  6 %  52 %  39 %  30 %  3 % | 36  99  96  22  55  48  27  8 | | 33 %  92 %  89 %  20 %  51 %  44 %  25 %  7 % | 0.83  0.028  0.55  0.065  1.00  0.69  0.65  0.69 |
| **Treatments at admission**  Anticoagulants  Antiplatelet agent  PPI before admission  **Treatments during ICU**  Enteral nutrition  Intravenous nutrition | 10  6  24  5  4 | 30 %  18 %  73 %  15 %  12 % | 26  15  45  45  26 | | 24 %  14 %  42 %  43 %  27 % | 0.65  0.65  0.003  0.004  0.15 |
| **GIB characteristics**  Upper GIB  Delay ICU – GIB*, days*  Melena  Hematochezia  Hematemesis | 20  0  15  13  13 | 61 %  [-2 – 0]  45 %  39 %  39 % | 83  0  75  38  29 | | 77 %  [-2 – 2.5]  69 %  35 %  27 % | 0.076  0.57  0.022  0.68  0.19 |
| **Biology during GIB**  Thrombopenia < 50 G/L  Hemoglobin (nadir)  PT  ACT  Lactatemia | 14  7.2  72.5  1.12  1.5 | 44 %  [6.4 – 7.4]  [61 – 80]  [1 – 1.37]  [0.9 – 1.7] | 42  6.4  68  1.19  2.55 | | 39 %  [5.4 – 6.9]  [56 – 76]  [0.9 – 1.5]  [1.4 – 4.3] | 0.68  0.003  0.10  0.45  0.0001 |
| **Organ failure**  Shock  Vasopressive drugs  Renal replacement therapy  Mechanical ventilation  SOFA score | 0  1  6  18  4 | 0 %  3 %  18 %  56 %  [1 – 7] | 64  54  38  92  6 | | 59 %  50 %  35 %  85 %  [4 – 14] | <0.0001  <0.0001  0.086  0.001  0.0008 |
| **Transfusion**  RBC units  PC units  FFP units | 2  0  0 | [2 – 4]  [0 – 8]  [0 – 0] | 6  8  0 | | [4 – 11]  [0 – 24]  [0 – 4] | <0.0001  0.018  <0.0001 |
| **Etiologies**  Other  Specific malignant lesion | 29  4 | 88 %  12 % | 76  32 | | 70 %  30 % | 0.066 |
| **Interventional care**  EGD, hemostatic procedure  Colonoscopy  CT angiography  Arterio-embolization  Hemostatic surgery | 12  11  1  0  0 | 36 %  33 %  3 %  0 %  0 % | 47  31  33  10  10 | | 44 %  29 %  31 %  9 %  9 % | 0.55  0.67  0.0008  0.12  0.12 |
| ICU stay*, days*  Deaths at day 90 | 2  6 | [1 – 5]  18 % | 6  48 | | [3 – 12]  44 % | <0.0001  0.008 |

*BMI: body mass index, PPI: proton pump inhibitor, ICU: intensive care unit, GIB: gastrointestinal bleeding, PT: prothrombin time, ACT: activated coagulation time, SOFA: sequential organ failure assessment, RBC: red blood cell, FFP: fresh frozen plasma, PC : platelet concentrates, GVH: graft versus host, EGD: esophagogastroduodenoscopy, CT: computed tomography.*

**Table S3: Univariate analysis: risk factors associated with mortality**

| **Characteristics** | Survivors at day 90 | | Deaths at day 90 | | p value |
| --- | --- | --- | --- | --- | --- |
|  | N=87 | % or med [IQR] | N=54 | % or med [IQR] |  |
| **Generalities**  Age  Male gender  Weight  BMI | 60  53  70  24 | [50 – 69]  61 %  [57.7 – 84]  [21 – 29] | 55.5  36  72.75  25 | [45 – 68.7]  67 %  [65.5 – 82]  [22 – 29.5] | 0.50  0.59  0.26  0.21 |
| **Immunosuppression factor**  Hemopathy  Auto-immune disease  Solid organ transplant  HIV  Corticosteroids  Chemotherapy  Immunosuppressive drugs  Allograft | 57  8  11  18  37  37  22  1 | 66 %  9 %  13 %  21 %  43 %  43 %  25 %  1 % | 36  9  6  6  35  24  15  8 | 67 %  17 %  11 %  11 %  65 %  44 %  28%  15 % | 1.00  0.20  1.00  0.17  0.015  0.86  0.84  0.002 |
| **Treatments at admission**  Anticoagulants  Antiplatelet agent  PPI before admission  **Treatments during ICU**  Enteral nutrition  Intravenous nutrition | 19  17  43  21  13 | 22 %  20 %  49 %  25 %  16 % | 17  4  26  29  17 | 31 %  7 %  48 %  54 %  38 % | 0.12  0.12  1.00  0,001  0.008 |
| **GIB characteristics**  Upper GIB  Delay ICU – GIB, *days*  Melena  Hematochezia  Hematemesis | 55  0  55  34  25 | 63 %  [-2 – 0]  63 %  39 %  29 % | 48  0  35  17  17 | 89 %  [-1.75 – 4]  65 %  31 %  31 % | 0.0008  0.18  1.00  0.37  0.85 |
| **Biology during GIB**  Thrombopenia < 50 G/L  Hemoglobin (nadir)  PT  ACT  Lactatemia  Fibrinogen (g/l) | 31  6.6  71.5  1.11  1.9  3.13 | 36 %  [5.7 – 7.3]  [59 – 78.7]  [1 – 1.4]  [1.1 – 2.6]  [2.3 – 4.9] | 25  6.5  66  1.3  3.15  2.92 | 46 %  [5.5 – 7.2]  [54 – 76]  [1 – 1.7]  [1.4 – 4.4]  [1.7 – 4.7] | 0.29  0.60  0.083  0.021  0.009  0.19 |
| **Organ failure**  Shock  Vasopressive drugs  Renal replacement therapy  Mechanical ventilation  SOFA score  AIM65 score ≥ 2 | 29  25  22  64  4  58 | 33 %  29 %  25 %  74 %  [1.5 – 7.5]  67% | 35  30  22  46  11  47 | 65 %  57 %  41 %  85 %  [6 – 14]  87% | 0.0004  0.001  0.063  0.14  <0.0001  0.009 |
| **Transfusion**  RBC units  PC units  FFP units  Ratio FFP/RBC  Low  Moderate  High  Ratio PC/RBC  Low  Moderate  High | 5  4  0  71  7  5  39  5  40 | [2 – 8.5]  [0 – 15]  [0 – 2]  86%  8%  6%  46%  6%  48% | 6  8  0  29  13  7  15  6  29 | [3 – 10.75]  [0 – 24]  [0 – 4]  59%  27%  14%  30%  12%  58% | 0.49  0.07  0.041  0.003  0.12 |
| **Etiologies**  Specific malignant lesion  Other | 19  68 | 22 %  78 % | 17  37 | 31 %  69 % | 0.24 |
| **Interventional care**  EGD, hemostatic procedure  Colonoscopy  CT angiography  Arterio-embolization  Hemostatic surgery | 38  29  18  5  6 | 44 %  33 %  21 %  6 %  7 % | 21  13  16  5  4 | 39 %  24 %  30 %  9 %  7 % | 0.60  0.26  0.23  0.51  1.00 |
| Relapses  ICU stay*, days* | 20  4 | 34  [2 – 9.75] | 18  7 | 49  [4 – 14] | 0.20  0.007 |

*BMI: body mass index, PPI: proton pump inhibitor, ICU: intensive care unit, GIB: gastrointestinal bleeding, PT: prothrombin time, ACT: activated coagulation time, SOFA: sequential organ failure assessment, RBC: red blood cell, FFP: fresh frozen plasma, PC: Platelet concentrates, GVH: graft versus host, EGD: esophagogastroduodenoscopy, CT: computed tomography.*

**Table S4**

**Factors associated with mortality by multivariate analysis (including the AIM65 score)**

|  | OR | 95%CI | p value |
| --- | --- | --- | --- |
| AIM65 score≥ 2 | 2.26 | 0.83- 6.18 | 0.11 |
| Upper GIB | 4.97 | 1.75-14.16 | 0.00 |
| Long-term corticosteroids therapy | 3.10 | 1.40-6.80 | 0.01 |
| Albumin (per 5 g/l increase) | 0.56 | 0.36-0.87 | 0.01 |

**Table S5**

**Factors associated with mortality by multivariate analysis (including the FFP/RBC ratio)**

|  | OR | 95%CI | p value |
| --- | --- | --- | --- |
| Shock | 0.95 | 1.15-5.75 | 0.02 |
| Upper GIB | 1.54 | 1.59-13.6 | 0.005 |
| Long-term corticosteroids therapy | 1.15 | 1.38-7.24 | 0.006 |
| Albumin (per 5 g/l increase) | 0.65 | 0.33-0.82 | 0.004 |
| FFP/RBC ratio | 0.53 | 0.80-3.64 | 0.17 |

**Figure S1**

**Case-control analysis : Love plot comparing SMD (standardized Mean Differences) before and after matching**
